# Psychotherapy Dropout: Using the Adolescent Psychotherapy Q-Set to Explore the Early In-Session Process of Short-Term Psychodynamic Psychotherapy

**DOI:** 10.3389/fpsyg.2021.708401

**Published:** 2021-10-22

**Authors:** Hanne Gotaas Fredum, Felicitas Rost, Randi Ulberg, Nick Midgley, Agneta Thorén, Julie Fredrikke Dalen Aker, Hanna Fam Johansen, Lena Sandvand, Lina Tosterud, Hanne-Sofie Johnsen Dahl

**Affiliations:** ^1^Department of Psychology, University of Oslo, Oslo, Norway; ^2^Portman Clinic, The Tavistock and Portman NHS Foundation Trust, London, United Kingdom; ^3^Division of Mental Health and Addiction, Institute of Clinical Medicine, University of Oslo, Oslo, Norway; ^4^Vestfold Hospital Trust, Division of Mental Health, Research Unit, Tønsberg, Norway; ^5^Department of Psychiatry, Diakonhjemmet Hospital, Oslo, Norway; ^6^Anna Freud National Centre for Children and Families, The Kantor Centre of Excellence, London, United Kingdom; ^7^The Erica Foundation, Stockholm, Sweden

**Keywords:** adolescence, dropout, interaction structures, treatment process, psychodynamic psychotherapy, Q-analysis

## Abstract

Research suggests that short-term psychodynamic psychotherapy (STPP) is an effective treatment for depression in adolescence, yet treatment dropout is a major concern and what leads to dropout is poorly understood. Whilst studies have begun to explore the role of patient and therapist variables, there is a dearth of research on the actual therapy process and investigation of the interaction between patient and therapist. This study aims to address this paucity through the utilisation of the Adolescent Psychotherapy Q-set (APQ) to examine the early treatment period. The sample includes 69 adolescents aged 16–18 years with major depressive disorder receiving STPP as part of the First Experimental Study of Transference Work–in Teenagers (FEST-IT) trial. Of these, 21 were identified as dropouts and were compared to completers on pre-treatment patient characteristics, symptomatology, functioning, and working alliance. APQ ratings available for an early session from 16 of these drop out cases were analysed to explore the patient-therapist interaction structure. Results from the Q-factor analysis revealed three distinct interaction structures that explained 54.3% of the total variance. The first described a process of mutual trust and collaboration, the second was characterised by patient resistance and emotional detachment, the third by a mismatch and incongruence between therapist and adolescent. Comparison between the three revealed interesting differences which taken together provide further evidence that the reasons why adolescents drop out of therapy vary and are multidimensional in nature.

## Introduction

Depressive disorders are among the main causes of long-term disability worldwide (James et al., 2018). Three quarters of adults with mental illness first experience symptoms before the age of 25 (Atkinson, 2018). Over the past decade, we have seen a striking increase in mood disorders and suicide-related outcomes among adolescents (Collishaw, 2015; Mojtabai et al., 2016; Atkinson, 2018; Twenge et al., 2018). This suggests that the provision of adequate treatment at that age is paramount. Reducing the duration and preventing recurrence and relapse of depression can lessen the burden on the young person, their family, and society at large (Goodyer et al., 2017) as well as reducing the high prevalence of depression in adulthood. Faced by this situation, attempts have been made to make mental health services more accessible and responsive to the particular needs of young people (Jurewicz, 2015). Yet a crucial challenge remains, which is that adolescents tend to report fewer positive attitudes toward help seeking than adults (Radez et al., 2021) and tend to show high rates of premature dropout from psychological treatments (Warnick et al., 2012; de Haan et al., 2013).

Short-term psychodynamic psychotherapy (STPP) is effective for treating depression in adults (Leichsenring et al., 2004; Abbass et al., 2014; Steinert et al., 2017), and there is growing evidence that it may be beneficial for adolescents too (Abbass et al., 2013; Midgley et al., 2021). STPP is an umbrella term that captures a range of brief psychodynamic/psychoanalytic therapies that share common goals and processes (Malda Castillo et al., 2020). It usually comprises up to 30 weekly sessions and the focus of STPP goes beyond symptom reduction. It includes working on the patients’ emotional, relational, and behavioural patterns, exploring how these patterns relate to past experiences and are expressed in ongoing relationships, and promoting the restructuring of defences, improved interpersonal functioning and regulation of affect (Kenny, 2016). Manualised approaches include the Intensive Short-Term Dynamic Psychotherapy (ISTDP; Davanloo, 1999) and Short-Term Psychoanalytic Psychotherapy for Adolescents with Depression (Cregeen et al., 2017). In both of these psychodynamic approaches, it is theorised that the patient’s transference (e.g., the patient’s past relational history, affective experiences, and attachment patterns) influences the ongoing interaction between patient and therapist and is one focus for therapeutic interventions, by means of “transference work” (TW).

The largest and most robust randomised controlled trial to test the efficacy of STPP for depressed adolescents was the Improving Mood with Psychoanalytic and Cognitive Therapies Study (IMPACT; Goodyer et al., 2017). It included 465 adolescents diagnosed with moderate and severe depression and compared STPP to CBT and a manualised Brief Psychosocial Intervention (BPI). The study found STPP to be equally efficacious in reducing depressive symptoms as CBT and BPI. Most importantly, adolescents showed sustained treatment effects over the one-year follow-up after treatment ended (Goodyer et al., 2017). These findings led the National Institute for Health and Care Excellence guideline for depression in children and young people (NICE, 2019) to recommend STPP as one treatment option to be offered to this population, thus increasing patient choice, especially in the case where young people were initially unresponsive to CBT.

Whilst the IMPACT study helped to demonstrate the effectiveness of STPP for depressed adolescents, questions remained about mechanisms of change. Addressing the need to investigate whether specific psychotherapeutic techniques influence outcome, the FEST-IT study (Ulberg et al., 2012, 2021) randomised 69 depressed adolescents to 28 sessions of STPP with or without TW. In the FEST-IT study, TW is defined as the therapist working directly with the client-therapist relationship as it is manifested in the therapy setting, as compared to a therapy in which the therapist may be aware of transference dynamics but does not explicitly address these in the clinical setting. This made it possible to examine whether TW, often considered a key feature of STPP, is an essential element of effective STPP for adolescents.

The FEST-IT study found that individuals in both treatment arms improved in terms of the main outcome measure, the Psychodynamic Functioning Scales (PFS), but that those who received TW had significantly better outcomes in terms of depression severity than those who did not receive TW, changes that were sustained in the long-term. The authors concluded that the psychodynamic approach led to improvements in family relations, insight, affect regulation, and adaptive capacity overall. However, the particular attention to thoughts and emotions of the adolescent in relation to the therapist contributed to an additional decrease in depression symptoms and severity (Ulberg et al., 2021).

Whilst these findings provide important evidence and insight into the specific mechanisms of change, a major challenge for psychotherapy research trials and for clinicians alike is therapy dropout. It has implications for both service providers and the individuals who drop out. Whilst it wastes time and potentially resources in an already stretched national health care system with long waiting times (Bohart and Greaves Wade, 2013), it may also prolong the suffering of the individuals who end their treatment prematurely (Hansen et al., 2002). However, whilst studies have linked dropout to poorer outcomes in adult depression (Saatsi et al., 2007; Saxon and Barkham, 2012), the link between the two in youth depression is unclear (O’Keeffe et al., 2019). Overall, studies indicate that between 28% and 75% of young people drop out of therapy, often leaving suddenly within the first few sessions (de Haan et al., 2013). However, treatment dropout is less studied in psychodynamic oriented treatments compared to other therapy approaches (Gabbard, 2009).

In order to understand the causes better, research has aimed at identifying pre-treatment client factors that may reliably predict psychotherapy dropout among young people. Kazdin et al. (1995) proposed a risk factor model, which includes low socio-economic status, being brought up by a single parent, being less educated, experiencing high number of adverse life events, and problems at home. The latest meta-analytic study, however, found less agreement between studies in terms of the predictability of these variables (de Haan et al., 2013). The most reliable predictor of premature ending of treatment for adolescents so far has been the absence of a good therapeutic alliance early in treatment (Robbins et al., 2006; de Haan et al., 2013; O’Keeffe et al., 2018), confirming findings found in adult populations (see Philips et al., 2018 for a summary). However, alliance has mainly been assessed using self-report questionnaires and discrepant findings have been observed between patient-rated and therapist-rated alliance (de Haan et al., 2013; Ormhaug and Jensen, 2018).

In order to address the crucial question as to why adolescents drop out, O’Keeffe et al. (2019), utilising a mixed-methods design to examine qualitative data from the IMPACT study, identified three distinct drop out types found across different therapeutic approaches: dissatisfied, got-what-they-needed, and troubled dropouts. The dissatisfied adolescents stopped their treatment because they did not like the intervention and felt it could not address what they sought therapy for. The got-what-they-needed type stopped therapy because they felt they had improved and were not in need of further treatment – even if their therapist felt the work was not completed. The troubled adolescents stopped attending because life events caused instability, impacting on their capacity to engage with regular outpatient therapy. In a second study, O’Keeffe et al. (2020) found that the got-what-they-needed dropouts had similar alliance scores than treatment completers, whereas the dissatisfied dropouts showed poorer alliance with their therapists and had more unresolved alliance ruptures compared to both completers and got-what-they-needed dropouts. Most interesting, however, was that whilst therapists and patients of the got-what-they-needed dropouts had similar narratives about therapy, the dissatisfied dropouts had quite divergent narratives to their therapists. In particular, therapists of dissatisfied dropouts often seemed unaware of the things that the adolescent did not like about therapy and were more likely to explain their ending treatment as due to “resistance” to the painful work of therapy.

Few studies have focussed on the therapy process to shed light on the crucial question as to why adolescents drop out. One approach that may help to explore both specific therapy techniques and interpersonal interaction was developed by Jones (2000). Combining concepts such as enactment, intersubjectivity, and role-responsiveness, he proposed the existence of patterns in the interaction between therapist and patient that emerge consciously or unconsciously during the therapeutic process. He referred to these patterns as “interaction structures” and identified them as an essential part of the psychotherapy process leading to either facilitate or impede it (Jones and Ablon, 2005). To assess interaction structures more systematically, Jones (2000) developed the Psychotherapy Process Q-set (PQS). It consists of 100 items describing a range of patient and therapist activities (behaviours, attitudes, feelings, and experiences) and the nature of the interaction between both. The PQS has inspired the development of a Q-set suitable for child psychotherapy (CPQ; Schneider, 2004; Schneider et al., 2010), and more recently a Q-set tailored for adolescent psychotherapy (APQ; Calderón et al., 2017). In contrast to variable-centred questionnaires or structured interviews, the items in a Q-sort are rank-ordered in relation to each other to obtain a holistic composite description of whatever is being studied, in this case the therapy session. It thereby retains the complexity of various interdependent variables, including those that belong to the patient, the therapist, and their dynamic interaction (Rost et al., 2018; Rost, 2021). Subjecting Q-sorts to Q-factor analysis (Stephenson, 1953) allows for the identification of similarity or difference between whole sessions rather than between individual variables.

Whereas a few multiple-case and single-case studies have identified a number of interaction structures and linked these with outcome in adult psychotherapy (Ablon et al., 2011; Goodman et al., 2014; Serralta, 2016; Laskoski et al., 2019) and child psychotherapy (Goodman and Athey-Lloyd, 2011; Goodman, 2015; Ramires et al., 2017, 2020; Odhammar et al., 2019), there is only one study that explored these in adolescents (Calderón et al., 2019) and none in individuals of any age range who dropped out of treatment. Philips et al. (2018) utilised the PQS to explore the early psychotherapy process between completers and dropouts of six adult patients with a dual diagnosis who received mentalisation-based treatment (MBT). Although they did not explore differences in interaction structures, the comparison between the treatment process revealed significant differences. An interesting observation was that the therapists of those patients who subsequently dropped out, somewhat deviated from the MBT approach. They provided more advice, behaved in a teacher-like manner, interpreted others’ behaviour, and their own emotional conflicts intruded into the relationship. The patients were emotionally detached and talked about wanting to be separate. Those MBT therapists who treated completers on the other hand communicated clearly, perceived the process accurately and commented on changes in patients’ affect. The patients in turn could talk confidently about themselves, their issues, and interpersonal relationships.

The present study endeavoured to contribute to our understanding of the therapy process in adolescents who went on to drop out of therapy. Utilising the data from the First Experimental Study of Transference Work–in Teenagers (FEST-IT) trial, the first aim was to identify, describe and compare the adolescents that ended treatment prematurely on their pre-treatment characteristics, symptomatology and functioning to those who did not drop out of STPP. Guided by previous research findings, we expected adolescents who dropped out to have poorer relational and intrapsychic functioning prior to treatment starting compared to treatment completers. No differences concerning other pre-treatment patient characteristics were expected. We hypothesised that dropouts would have poorer alliance scores and display lower motivation early in the treatment process compared to completers. The final aim was to examine whether particular interaction structures characterised the psychotherapy process of the early sessions of those who subsequently dropped out. Given the lack of previous research guiding specific predictions, we adopted an explorative approach to address this research question.

## Materials and Methods

### Setting

This study draws on data from the FEST-IT study (Ulberg et al., 2012, 2021). Sixty-nine adolescents with major depressive disorder (MDD) were randomised to Short Term Psychodynamic Psychotherapy (STPP) either with (*n* = 39) or without (*n* = 30) TW for 28 once-weekly 45 minute sessions. Participants were recruited from private practices and public child and adolescent outpatient health care services in the Oslo area and (the former) Vestfold County of the South-Eastern Health region in Norway. Depression and other Axis I diagnoses were assessed with the Mini International Neuropsychiatric Interview (M.I.N.I.; Sheehan et al., 1998) and the Structured Interview for DSM-IV Personality (SIDP-IV; Pfohl et al., 1997) was used to capture Axis II diagnostics. Exclusion criteria were bipolar depression, learning difficulties, pervasive developmental disorders, psychosis, and substance addiction. Diagnostic and clinical interviews were completed before, after, and one-year after treatment ended. Symptom severity was self-reported at the same time points, as well as collected during therapy. A detailed description of these measures and time points can be found elsewhere (Ulberg et al., 2012). The treatment was based on STPP for Adolescents with Depression manualised by Cregeen et al. (2017). The manual outlines the importance of interpretation of unconscious conflicts, attachment theory, and the notion of inner working models. The interventions in both treatment arms were directed at exploring the adolescent’s interpersonal relationships as well as the thoughts and feelings that the adolescent possibly evades, and this calls for repetitive patterns of thoughts, feelings, and actions. In the treatment arm applying a moderate level of TW (i.e., one to three per session), the therapists prescribed additional interventions that (a) addressed the dynamic of the patient-therapist relationship in the here-and-now; (b) instigated exploration of thoughts and feelings regarding the therapy and the therapist; and (c) drew attention to direct manifestations of transference and linked repetitive interpersonal patterns to transactions between patient and therapist. In the none-TW group, these interventions from the therapist were proscribed. All psychotherapy sessions were audio recorded.

### Participants

The patients were 57 female and 12 male adolescents between the age of 16–18 years (mean age 17.3). The therapists (*N* = 12) were experienced clinical psychologists or psychiatrists with a minimum of two years of formal education in psychodynamic psychotherapy and special training in psychotherapy with children and adolescents. In addition, they attended a 1-year training program to provide psycho dynamic psychotherapy both with a moderate level of transference interpretations and without transference interpretations. All therapists treated adolescents in both treatment modalities.

### Measures

#### Beck Depression Inventory-II

Beck Depression Inventory-II (BDI-II; Beck et al., 1996) is a widely used 21-item self-report questionnaire measuring depression severity with a range of scores from 0 to 63. It has shown to have good reliability and established validity in an adolescent population (Beck et al., 1996; Wang and Gorenstein, 2013).

#### Montgomery and Åsberg Depression Rating Scale

Montgomery and Åsberg Depression Rating Scale (MADRS; Montgomery and Asberg, 1979) is a widely used screening instrument for observer-rated depression severity with well-established reliability and validity (Svanborg and Åsberg, 2001). The MADRS was rated by one independent and blinded rater and the therapist in 30% of the patients. The intra-class correlation coefficient (ICC) for the MADRS single measure was 0.78 (Cl 0.58–0.9).

#### The Psychodynamic Functioning Scales

The Psychodynamic Functioning Scales (PFS; Høglend et al., 2000) is based on a psychodynamic clinical interview, and assesses levels of interpersonal functioning (quality of family relations and quality of friendships) and intrapsychic functioning (tolerance for affects, insight, and problem-solving capacity) on a scale rated from 1 to 100 with higher scores representing better functioning. The PFS subscales have demonstrated good inter-rater reliability in an adolescent population (Ness et al., 2018). The PFS was rated by three independent raters blind to treatment allocation. The ICC for the PFS was 0.82 (CI 0.73–0.91).

#### The Global Assessment of Functioning

The Global Assessment of Functioning (GAF; American Psychiatric Association [APA], 2002) is based on a semi-structured interview and attempts to quantify the overall functioning level of an individual. It seeks to measure how much a person’s symptoms affect psychosocial and occupational or educational functioning on a scale from 1 (severely impaired) to 100 (extremely high functioning).

#### Credibility/Expectancy Questionnaire

Credibility/Expectancy Questionnaire (CEQ; Borkovec and Nau, 1972) was used to measure global treatment expectancy before treatment started. Patients indicate their level of confidence in the treatment’s helpfulness on a single visual analog scale ranging from 0 = “pointless” to 50 = “moderate confidence” to 100 = “all problems will be resolved”. Its psychometric properties have been found to be reasonably good (Borkovec and Costello, 1993; Devilly and Borkovec, 2000) and it has been used in studies examining outcome and working alliance (e.g., Meyer et al., 2002; Vogel et al., 2006).

#### The Working Alliance Inventory-Short Revised

The Working Alliance Inventory-Short Revised (WAI-SR; Hatcher and Gillaspy, 2006) is a 12-item self-report questionnaire measuring the strength of the therapeutic alliance. Based on Bordin’s (1979) conceptualisation, it incorporates agreement on the goals, on tasks, and on the emotional bond. The Norwegian version is rated on a seven-point Likert scale ranging from *never* (1) to *always* (7). Higher scores indicate better alliance with a total mean score ranging from 1 to 7. It was rated after the third session by both patient and therapist. It has shown to have good reliability and validity (Hatcher and Gillaspy, 2006; Munder et al., 2010).

#### A Bespoke Motivation Scale

A bespoke Motivation Scale (TMS) was used to measure adolescents’ motivation and willingness to cooperate in therapy, rated by their therapists after the third session using a visual analogous rating scale with anchored endpoints, ranging from 1 (The patient displayed great resistance and would not cooperate at all) to 10 (The patient displayed high commitment and partaking).

#### The Adolescent Psychotherapy Q-Set

The Adolescent Psychotherapy Q-set (APQ; Calderón et al., 2017) is a 100-item Q-sort measure describing the patient (e.g., “Young person feels sad or depressed”) and the therapist (e.g., “Therapist attends to young person’s current emotional states”) activity and the interaction between them (e.g., “Young person resists therapist’s attempts to explore thoughts, reactions, or motivations related to problems”). Following a fixed distribution, the rating procedure involves rank-ordering the 100 items based on their applicability of the particular therapy session from 1 (*extremely uncharacteristic*) to 9 (*extremely characteristic*). The midpoint 5 (*relatively neutral*) contains the items that are unimportant in describing the session. The fixed distribution of items is 5 × 1, 8 × 2, 12 × 3, 16 × 4, 18 × 5, 16 × 6, 12 × 7, 8 × 8, and 5 × 9. The coding manual (Calderón et al., 2014) provides definitions of every item with examples to guide the process. The APQ has demonstrated good validity and reliability (Calderón et al., 2017). The APQ was rated by four trained researchers after listening to the audio-taped therapy sessions and carried out using an electronic version (Dawson, 2013). Each rating took about 2 to 3 hours. Rater reliability was ascertained before rating during an extensive two-day training with the developer and inter-rater reliability was monitored carefully throughout. All raters were blind to the study arm allocation and inter-rater reliability (IRR) was assessed with the ICC, using a two-way mixed consistency model (Shrout and Fleiss, 1979). Inter-rater reliability for session three was assessed in 30% with all ICCs > 0.60 which can be considered satisfactory (Cicchetti, 1994).

### Procedure

#### Patient and Session Selection

As per the FEST-IT protocol drop out was defined as ending treatment any time up to the 12th session. Main categorisations of dropout usually include duration of the therapy (i.e., when the adolescent in a study ends treatment before the pre-defined cut off) and therapist judgement of whether the treatment ending is a dropout (Edbrooke-Childs et al., 2021). We do not have information as to whether therapists deemed the patients as drop out or not. After session three, both therapist and patient rated the WAI, and the therapist rated the patient’s level of motivation. We wanted as many perspectives on the process as possible and chose session three for further process analysis with the APQ. 21 patients meeting the dropout criteria were identified, indicating a dropout rate of 30%. However, as both data and recordings of sessions were unavailable for five patients, the total sample size for the process data analysis with the APQ was 16.

### Data Analysis

To answer our first research questions, we used descriptive statistics and frequencies to describe and compare the dropouts and completers. Statistical analyses were carried out in IBM SPSS Statistics (Version 27). Mean differences were analysed using independent sample *t*-tests. A *t* value of ±1.96 was significant at the *p* < 0.05 level. Differences of categorical variables were analysed using chi-square statistics. *Post hoc* tests included the comparison of specific cells and calculation of adjusted residuals. A *post hoc z* score of ± 1.96 was significant at the *p* < 0.05 level.

To investigate the psychotherapy process, we first examined the general description of the third session of adolescent dropouts by obtaining the most and least characteristics APQ items. These were calculated by aggregating the ratings of the 16 sessions and rank-ordering the means. To explore whether particular interaction structures can be identified among the sessions of the adolescent dropouts, we secondly subjected all APQ ratings of session three (*N* = 16) to Q-factor analysis. Principal component analysis was used and as there was no theoretical reason to assume complete independence of the resulting factor structure (Watts and Stenner, 2012), Promax rotation with Kaiser normalisation was used to rotate the factors to produce a final oblique solution. Following recommendations by Brown (1980), we combined the examination of statistical criteria with a thorough exploration of its theoretical meaningfulness in order to determine the final number of factors to be extracted. As such various factor solutions were quantitatively and qualitatively examined before settling on a final solution. Statistical criteria included the scree plot, percentage of variance explained, the Kaiser-Guttman criterion (to extract factor with an eigenvalue of >1) and Humphrey’s rule to accept those factors that have two or more significant factor loadings. We calculated that in this study factor loadings needed to be ≥0.35 to be significant at the 0.01 level (Brown, 1980). Significant Q-sorts that loaded on one factor only were weighted and merged to reveal the level of agreement each statement carries within the identified interaction structures (Valenta and Wigger, 1997). Significant factor scores were subsequently standardised (transformed into *z* scores) and applied to its initial ranking system. The final step consisted of an inspection and comparison of the patterns found in the items of each factor array, and a name was chosen for each factor to denote the most defining and differentiating aspect of the interaction structure (IS) identified. As such, particular attention was paid to items with high rankings (9, 8, and 7; items that characterise the IS) and low ranking (1, 2, and 3; items that do not characterise the IS). Q-factor reliability was assessed calculating the Cronbach alpha coefficient with α ≥ 0.8 suggesting adequate internal consistency (Fleiss, 1981). Items with a negative factor loading were reversed for that purpose.

### Ethical Considerations

Ethical approval for the FEST-IT study was granted by the Regional Committees for Medical and Health Research Ethics (REC) (REK: 2011/1424 FEST- IT). Fully informed and written consent was sought from all participants prior to entering the trial. To ensure confidentiality, participants were assigned a pseudonym, raters only assessed the sessions they had to code and any identifiable information about the therapists and the adolescents were changed or removed. Trial registration: ClinicalTrials.gov Identifier: NCT01531101

## Results

### Identification of Dropout and Pre-treatment Comparison With Completers

Table 1 displays the descriptive statistics and pre-treatment clinical and function indices for those who completed treatment and those who dropped out. As expected, no statistically significant differences were found regarding any demographic, clinical and functioning indices (all *p’s* and *X*^2^ > 0.5) except for number of personality disorder criteria as measured with the SIDP-IV (*t* = −218, *p* = 0.033). Frequency of a comorbid eating disorder was diagnosed in 4% of the completers only and high risk of suicide was also only reported in completers. Patient-rated treatment expectancy mean scores indicated high expectations, and no statistically significant differences between the two groups were found (*t* = 0.930, *p* = 0.357). Contrary to expectations, analysis of the therapeutic alliance, both total score and all three sub-scales, revealed no statistically significant differences between the two groups (all *p* > 0.5), as well as no statistically significant difference in the therapist- compared to patient-rating (all *p* > 0.5). Regarding therapist-rating of the adolescent’s level of treatment motivation, however, those who dropped out were rated statistically significantly lower after the third session compared to those who completed (*t* = −2.460, *p* = 0.02). Overall, 21 adolescents dropped out after attending on average six sessions, those that completed attended on average 24 sessions. Whilst 60% of the completers received STPP with TW, amongst those who dropped out 48% received STPP with TW, however, this difference was not statistically significant (*X*^2^_(1)_ = 0.97, *p* = 0.32).

**TABLE 1:** Pre-treatment characteristics, working alliance, number of sessions, and randomisation group for treatment completers and dropouts.

	**Completers (*n* = 48)**	**Dropouts (*n* = 21)**
Age (M/SD)	17.3 (0.7)	17.3 (0.7)
% Female	41 (85%)	16 (76%)
**Treatment**		
% STPP with transference work	29 (60%)	10 (48%)
Number of sessions attended (M/SD)	24 (4.9)	6 (3.3)
**% Co-morbid Axis-I disorders (MINI)**		
Anxiety disorders	23 (48%)	10 (48%)
Post-traumatic stress disorder	1 (2%)	1 (5%)
Eating disorders	2 (4%)	0
**Axis-II disorders (SIDP-IV)**		
Number PD criteria (M/SD)	13.4 (9.05)	9.7 (5.7)*
**% Suicide risk**		
No risk	41 (73%)	16 (76%)
Moderate risk	4 (8%)	5 (24%)
High risk	3 (6%)	−

**Level of depression and functioning**	**M (SD)**	**M (SD)**

BDI	28.56 (9.01)	28.75 (9.56)
MADRS	23.22 (6.10)	21.60 (6.10)
GAF	59.77 (5.50)	58.77 (4.83)
PFS	60.37 (60.37)	57.95 (2.27)
Family	62.13 (8.63)	59.62 (9.21)
Friendship	64.02 (8.04)	62.57 (8.96)
Affect tolerance	56.42 (5.33)	54.67 (6.91)
Insight	59.06 (6.42)	54.76 (9.62)
Problem-solving/Adaptive capacity	60.15 (5.33)	58.14 (6.88)
Expectations	6.42 (1.99)	6.83 (1.46)

**Working Alliance and Motivation rated after session 3**	**M (SD)**	**M (SD)**

WAI Patient-rated Total	5.39 (0.81)	4.89 (1.20)
Goal	5.34 (0.96)	4.93 (1.58)
Task	5.46 (0.82)	4.88 (1.29)
Bond	5.37 (0.81)	4.86 (1.43)
WAI Therapist-rated Total	4.80 (0.95)	4.36 (0.91)
Goal	4.55 (1.15)	3.97 (1.11)
Task	4.85 (0.91)	4.35 (0.92)
Bond	5.01 (1.07)	4.75 (0.94)
Patient motivation (Therapist-rated TMS)	6.60 (1.99)	4.77 (2.51)

*p < 0.05. PD = Personality Disorder. BDI = Beck Depression Inventory-II; MADRS = Montgomery and Åsberg Depression Rating Scale; GAF = The Global Assessment of Functioning; PFS = The Psychodynamic Functioning Scales; CEQ = Credibility/Expectancy Questionnaire; WAI = The Working Alliance Inventory-Short Revised; TMS = Motivation Scale.

### General Description of the Psychotherapy Process of Dropouts

The ten most characteristic and ten least characteristic APQ items of the early therapy session were identified to describe the psychotherapy process for the adolescents that dropped out in general terms. These are displayed in full in Table 2. In brief, there seems to be interactions between an active therapist trying to engage the adolescent through asking to elaborate on feelings and symptoms, and an adolescent that accepts the therapist’s comments and observations, but without much curiosity or strong emotional engagement. The adolescent speaks extensively about feelings of sadness or low mood and is preoccupied with questions of self-identity and interpersonal relationships. The therapist is seeking to make sense of the adolescent’s experience but does not challenge often-expressed overgeneralisations or absolute beliefs, and the adolescent struggles to engage with their own thoughts and ideas.

**TABLE 2:** The ten most and ten least characteristic items of the treatment process of dropouts.

**APQ Item**	**Mean**
**Most characteristic**	
31. Therapist asks for more information or elaboration	7.688
9. Therapist works with young person to try to make sense of experience	7.563
54. Young person is clear and organised in self-expression	7.188
97. Therapist encourages reflection on internal states and affects	7.063
94. Young person feels sad or depressed	6.938
73. Young person discusses and explores current interpersonal relationships	6.813
35. Self-image is a focus of the session	6.750
39. Therapist encourages young person to reflect on symptoms	6.750
37. Therapist remains thoughtful when faced with young person’s strong affect or impulses	6.313
56. Material from a prior session is discussed	6.313
**Least characteristic**	
72. Young person demonstrates lively engagement with thoughts and ideas	3.750
71. Therapist challenges over-generalised or absolute beliefs	3.688
13. Young person is animated or excited	3.625
42. Young person rejects therapist’s comments and observations	3.625
87. Young person is controlling of the interaction with therapist	3.438
52. Young person has difficulty with ending of sessions	3.313
88. Young person fluctuates between strong emotional states during the session	3.063
5. Young person has difficulty understanding therapist’s comments	2.938
23. Young person is curious about the thoughts, feelings, or behaviour of others	2.875
67. Young person finds it difficult to concentrate or maintain attention during the session	2.813

### Identification of Interaction Structures

Q-factor analysis yielded three statistically sound and conceptually interpretable Q-factors (interaction structures) that together explained 54.3% of the total variance. Overall, three sessions were identified as confounders; one session did not reach the statistically significant level and two sessions loaded significantly onto two factors. Hence, 13 out of the 16 sessions were included in the analysis. Tables 3–5 displays the defining items with their respective factor loadings (converted into *z* scores) and ranking for each of the three Q-factors (interaction structures; IS). Q-factor 1, which was made up of five sessions and explained 25.3% of the variance, was named “***Mutual trust, collaboration, and the exploration of emotions.”*** Overall, 33 APQ items describe this IS with a high internal consistency (α = 0.914). Six sessions made up Q-factor 2 that added 20.1% to the total variance. This IS was named “***Resistance and emotional detachment***.***”*** 33 APQ items best describe this IS with excellent factor reliability (α = 0.942). Finally, Q-factor 3, which was made up of two sessions and added a further 8.9% to the total variance was termed “***Mismatch and incongruence in perception and communication***.***”*** This IS was best described by 29 APQ items with sound internal consistency (α = 0.881).

**TABLE 3:** Interaction structure 1: “Mutual trust, collaboration and the exploration of emotions.”

**APQ Item**	***Z*-score**	**Rank**
15. Young person does not initiate or elaborate topics	–2.29	1
44. Young person feels wary or suspicious of the therapist	–1.99	1
42. Young person rejects therapist’s comments and observations	–1.97	1
58. Young person resists therapist’s attempts to explore thoughts, reactions, or motivations related to problems	–1.91	1
35. Self-image is a focus of the session	1.90	9
9. Therapist works with young person to try to make sense of experience	1.90	9
14. Young person does not feel understood by therapist	–1.90	1
97. Therapist encourages reflection on internal states and affects	1.78	9
6. Young person describes emotional qualities of the interactions with significant others including therapist	1.72	8
92. Young person’s feelings or perceptions are linked to situations or behaviour of the past	1.69	8
50. Therapist draws attention to feelings regarded by young person as unacceptable	1.63	8
17. Therapist actively structures the session	–1.55	2
66. Therapist is directly reassuring	–1.54	2
40. Young person communicates with affect	1.36	8
46. Therapist communicates with young person in a clear, coherent style	1.24	8
84. Young person expresses angry or aggressive feelings	1.24	8
27. Therapist offers explicit advice and guidance	–1.23	2
26. Young person experiences or expresses troublesome (painful) affect	1.23	7
52. Young person has difficulty with ending of sessions	–1.04	2
85. Therapist encourages young person to try new ways of behaving with others	–0.96	3
32. Young person achieves a new understanding	0.93	7
82. Therapist adopts a problem-solving approach with young person	–0.90	3
49. There is discussion of specific activities or tasks for the young person to attempt outside of session	–0.86	3
19. Young person explores loss	0.83	7
1. Young person expresses, verbally or non-verbally, negative feelings toward therapist	–0.83	3
28. Young person communicates a sense of agency	–0.82	3
96. Therapist attends to the young person’s current emotional states	0.79	7
89. Therapist makes definite statements about what is going on in the young person’s mind	–0.77	3
12. Silences occur during the session	–0.75	3
62. Therapist identifies a recurrent pattern in young person’s behaviour or conduct	0.74	7
100. Therapist draws connections between the therapeutic relationship and other relationships	–0.72	3
29. Young person talks about wanting to be separate or autonomous from others	–0.72	3
86. Therapist encourages reflection on the thoughts, feelings and behaviour of significant others	–0.69	3

**TABLE 4:** Interaction structure 2: “Resistance and emotional detachment.”

**APQ Item**	***Z*-score**	**Rank**
39. Therapist encourages young person to reflect on symptoms	2.15	9
53. Young person discusses experiences as if distant from his feelings	2.15	9
73. Young person is committed to the work of therapy	–2.14	1
23. Young person is curious about the thoughts, feelings, or behaviour of others	–2.09	1
15. Young person does not initiate or elaborate topics	2.03	9
24. Young person demonstrates capacity to link mental states with action or behaviour	–2.01	1
40. Young person communicates with affect	–2.00	1
58. Young person resists therapist’s attempts to explore thoughts, reactions, or motivations related to problems	1.91	9
13. Young person is animated or excited	–1.85	1
95. Young person feels helped by the therapy	–1.72	2
88. Young person fluctuates between strong emotional states during the session	–1.56	2
25. Young person speaks with compassion and concern	–1.42	2
32. Young person achieves a new understanding	–1.40	2
12. Silences occur during the session	1.34	8
93. Therapist refrains from taking position in relation to young person’s thoughts or behaviour	1.31	8
68. Therapist encourages young person to discuss assumptions and ideas underlying experience	1.26	8
78. Young person seeks therapist’s approval, affection or sympathy	–1.23	2
47. When the interaction with young person is difficult, therapist accommodates in an effort to improve relations	1.22	7
74. Humour is used	–1.03	3
7. Young person is anxious or tense	1.02	7
50. Therapist draws attention to feelings regarded by young person as unacceptable	–0.99	3
61. Young person feels shy or self-conscious	0.96	7
44. Young person feels wary or suspicious of the therapist	0.92	7
26. Young person experiences or expresses troublesome (painful) affect	–0.80	3
48. Therapist encourages independence in the young person	0.77	7
81. Therapist reveals emotional responses	–0.72	3
10. Young person displays feelings of irritability	0.69	7
76. Therapist explicitly reflects on own behaviour, words or feelings	–0.69	3
60. Therapist draws attention to young person’s characteristic ways of dealing with emotion	–0.68	3
29. Young person talks about wanting to be separate or autonomous from others	0.68	7
6. Young person describes emotional qualities of the interactions with significant others including therapist	–0.68	3
8. Young person expresses feelings of vulnerability	–0.66	3
1. Young person expresses, verbally or non-verbally, negative feelings toward therapist	0.62	7

**TABLE 5:** Interaction structure 3: “Mismatch and incongruence in perception and communication.”

**APQ Item**	***Z*-score**	**Rank**
93. Therapist refrains from taking position in relation to young person’s thoughts or behaviour	–2.23	1
16. Young person fears being punished or threatened	1.96	9
17. Therapist actively structures the session	1.95	9
71. Therapist challenges over-generalised or absolute beliefs	–1.95	1
53. Young person discusses experiences as if distant from his feelings	–1.68	1
99. Therapist raises questions about young person’s view	–1.68	1
19. Young person explores loss	–1.68	1
56. Material from a prior session is discussed	1.67	8
82. Therapist adopts a problem-solving approach with young person	1.67	8
55. Young person feels unfairly treated	1.41	8
66. Therapist is directly reassuring	1.41	8
49. There is discussion of specific activities or tasks for the young person to attempt outside of session	1.40	8
25. Young person speaks with compassion and concern	1.40	8
20. Young person is provocative, tests limits of therapy relationship	–1.40	2
4. Young person’s treatment goals are discussed	1.39	7
3. Therapist’s remarks are aimed at facilitating young person’s speech	–1.12	2
57. Therapist explains rationale behind technique or approach to treatment	1.12	7
24. Young person demonstrates capacity to link mental states with action or behaviour	1.12	7
91. Young person discusses behaviours or preoccupations that cause distress or risk	1.10	7
97. Therapist encourages reflection on internal states and affects	–0.85	3
76. Therapist explicitly reflects on own behaviour, words or feelings	0.85	7
35. Self-image is a focus of the session	–0.85	3
41. Young person feels rejected or abandoned	0.84	7
27. Therapist offers explicit advice and guidance	0.84	7
75. Therapist pays attention to young person’s feelings about breaks, interruptions or endings in therapy	–0.84	3
65. Therapist restates or rephrases young person’s communication in order to clarify its meaning	0.83	7
73. Young person is committed to the work of therapy	0.83	7
10. Young person displays feelings of irritability	–0.83	3
61. Young person feels shy or self-conscious	–0.83	3

### Further Exploration of the Interaction Structures

To facilitate the interpretation and sense-making of the three IS, differences in pre-treatment demographic and clinical information of the adolescents were considered. Due to the small and unequal sample sizes, no test statistic was calculated. Frequencies and mean scores are displayed in Table 6. Overall, those adolescents whose sessions were characterised by IS 2 attended less sessions with an average of 5.3 sessions compared to those in IS 1 who attended 6.8 sessions and those in IS 3 who attended 8.5 sessions on average. The three groups also differed in terms of treatment group allocation. Whilst 80% of those in IS 1 received STPP with TW, 67% of those in IS 2 received STPP without TW. The two adolescents in IS 3 were allocated to one arm each. The adolescents did not differ in terms of age. Only IS 2 had male adolescents among them; IS 1 and IS 3 were entirely made up of females. In terms of Axis-I disorders, all adolescents in IS 2 had a co-morbid anxiety disorder and an additional 17% had a diagnosis of PTSD, whilst only 20% of those in IS 1 and 50% of those in IS 3 had an anxiety disorder and none were diagnosed with PTSD. In terms of co-morbid personality disorder criteria, those in IS 1 and IS 3 had on average less criteria compared to the IS 2; 8 versus 14.3. Adolescents in IS 2 furthermore differed from those in IS 1 and IS 3 in that their depression scores both on the BDI and the MADRS fall into the moderate and mild range, respectively, whilst the scores for IS 1 and IS 3 fell into the severe and moderate depression ranges. Although all adolescents show on average some impairment in family and friendship relations, insight, affect tolerance, and problem solving and adaptive capacity, as the average mid-range scores on the PFS sub-scales indicate, those in IS 2 fall one category lower on both the friendship, affect tolerance, and the insight sub-scale than IS 1 and IS 3. In terms of treatment expectancy, the individuals in each group did not seem to differ; the high mean score of each group indicates a great level of confidence in the treatment’s helpfulness. Finally, differences in mean scores on the patient and therapist-rated WAI can be observed. Although it is unknown whether differences are statistically significant, it is interesting to observe that the mean scores for those in IS 2 are lower on both patient and therapist-rated goal and task sub-scales compared to IS 1 and IS 3. There is a difference in mean scores between patient and therapist ratings on the WAI, particularly on the goal (6.4 versus 3.7) and task (6.0 versus 4.6) sub-scales, and the total score (5.9 versus 4.4) on IS 3. Overall, however, therapists and adolescents are quite similar when they rate the WAI. The therapist-rated motivation differs between IS 1 showing a relatively high motivation score at 6.2 compared to those in IS 2 at 2.2.

**TABLE 6:** Comparison of the three interaction structures in terms of patient and clinical characteristics, and working alliance in session three.

	**Interaction structure 1 (*n* = 5)**	**Interaction structure 2 (*n* = 6)**	**Interaction structure 3 (*n* = 2)**
Age (M/SD)	17.9 (1.0)	16.8 (0.6)	17.3 (1.8)
% Female	5 (100%)	3 (50%)	2 (100%)
**Treatment**			
% STPP with Transference work	4 (80%)	2 (33%)	1 (50%)
Number of sessions attended (M/SD)	6.8 (2.6)	5.3 (1.9)	8.5 (4.9)
**% Co-morbid Axis-I Disorders**			
Anxiety Disorders	1 (20%)	6 (100%)	1 (50%)
Post-traumatic stress disorder	0	1 (17%)	0
Eating disorders	0	0	0
**Axis-II disorders**			
SIDP-IV Number of PD criteria (M/SD)	8.0 (3.2)	14.3 (5.6)	8.0 (8.5)
**% Suicide risk**			
No risk	5 (100%)	2 (33%)	1 (50%)
Moderate risk	0	4 (67%)	1 (50%)
High risk	0	0	0

**Level of depression and functioning**	**M (SD)**	**M (SD)**	**M (SD)**

BDI	31.2 (4.8) severe	27.2 (13.7) moderate	34.5 (16.3) severe
MADRS	23.0 (4.2) moderate	18.7 (4.8) mild	27.5 (7.8) moderate
GAF	60.3 (3.0) moderate	60.5 (5.5) moderate	54.3 (0.9) moderate
PFS	59.6 (4.2)	56.3 (8.5)	59.3 (2.1)
Family	56.0 (8.0)	58.8 (10.7)	57.5 (0.7)
Friendship	64.6 (6.8)	60.1 (9.6)	64.0 (2.8)
Affect tolerance	58.0 (4.2)	51.2 (7.5)	56.5 (4.9)
Insight	60.2 (1.5)	47.5 (11.5)	58.5 (9.2)
Problem-solving/Adaptive capacity	59.0 (3.5)	57.2 (8.9)	60.0 (0)
Expectations	6.8 (1.3)	6.7 (1.2)	7.1 (1.9)
**Working alliance and motivation**			
WAI Patient-rated Total	5.2 (0.7)	4.1 (1.4)	5.9 (0.8)
Goal	5.1 (0.9)	3.9 (2.1)	6.4 (0.2)
Task	5.2 (0.4)	3.8 (1.3)	6.0 (0.4)
Bond	5.2 (1.0)	4.5 (1.9)	4.3 (1.1)
WAI Therapist-rated Total	4.8 (1.2)	3.8 (1.2)	4.4 (0.4)
Goal	4.4 (1.4)	3.5 (1.2)	3.7 (0.7)
Task	4.9 (1.1)	3.7 (0.9)	4.6 (0.2)
Bond	5.1 (1.4)	4.2 (0.4)	4.9 (0.2)
Motivation (Therapist-rated)	6.3 (2.3)	2.2 (1.0)	7.3*

*n = 1

*BDI = Beck Depression Inventory-II; MADRS = Montgomery and Åsberg Depression Rating Scale; GAF = The Global Assessment of Functioning; PFS = The Psychodynamic Functioning Scales; CEQ = Credibility/Expectancy Questionnaire; WAI = The Working Alliance Inventory-Short Revised; TMS = Motivation Scale.*

### Interaction Structures and Treatment Outcome

Table 7 displays the mean values for post-treatment data for the adolescents whose sessions fitted into each of the IS. Again, due to the very small sample of data available, no test statistic was calculated. Following mean values, those in IS 1 seemed to have become better, but one patient did not come for follow-up. They scored within the “mild depression” range on both the BDI and the MADRS at follow up (pre-treatment scores fell within “severe”/“moderate depression”), they scored 10 points higher (now 70 and outside clinical range) on the GAF, and almost outside the clinical range on the PFS. Those in IS 2 still scored within the “moderate depression” range and neither the scores on the GAF nor the PFS changed. The one girl for whom data was available for IS 3 moved out of depression, she also moved into the normal range on the GAF.

**TABLE 7:** Comparison of the three interaction structures in terms of depression and functioning post treatment.

	**Interaction structure 1 (*n* = 4)^a^**	**Interaction structure 2 (*n* = 4)^b^**	**Interaction Structure 3 (*n* = 1)^a^**
BDI	15.8 (11.6) mild	24.3 (18.2) moderate	12.0 none
MADRS	12.5 (7.0) mild	20.7 (6.1) moderate	4.0 normal
GAF	70.1 (6.0) normal	59.6 (6.2) moderate	72.8 normal
PFS Total score	66 (3.5)	59.5 (4.6)	66.6

*^*a*^Missing data for one participant. ^*b*^ Missing data from two participants.*

*BDI = Beck Depression Inventory-II; MADRS = Montgomery and Åsberg Depression Rating Scale; GAF = The Global Assessment of Functioning; PFS = The Psychodynamic Functioning Scales.*

## Discussion

Psychotherapy dropout among adolescents constitutes a major challenge for clinicians and is an indicator that depressed young people are not always getting optimal levels of therapeutic support. Whilst research has begun to explore possible risk factors in terms of client and therapist variables and the therapeutic alliance, very little research to date has focused on the exploration of the actual psychotherapy process to shed light onto what goes on in the therapeutic interaction for young people who decide to end their therapy. The aim of the study was to address this gap by firstly identifying dropouts among a sample of 69 adolescents who received STPP as part of an RCT and compare them to those who completed the treatment, in terms of pre-treatment characteristics, clinical and functioning severity. Secondly, by empirically examining (a) the therapeutic process of an early session in terms of their general description, and (b) as to its underlying interaction structures.

Results revealed that of the 69 adolescents in the FEST-IT study, 21 (30%) ended their treatment prematurely after having attended on average six sessions. The percentage of dropout appears similarly high to what was found in the IMPACT study (O’Keeffe et al., 2018). There were fewer receiving STPP with TW that dropped out percentage wise (60% versus 48%), but this difference was not statistically significant in this small sample. It is surprising that the effect of talking about the ongoing relationship does not seem to help those whose sessions were characterised by IS 2, who showed somewhat lower alliance. However, there is a debate about whether adolescents profit from transference work in psychodynamic therapy or not (Della Rosa and Midgley, 2017), with some suggesting that too much discussion of the adolescent-therapist relationship may run counter to the adolescent’s developmental need for a sense of autonomy. Transcripts of how the TW is delivered and received, might shed more light on these results. Ulberg et al. (2021) showed that on symptom measures of depression there was a positive effect of TW, yet this needs to be replicated in another population.

Confirming previous findings (O’Keeffe et al., 2018), patients in this study who dropped out of therapy were not found to differ with regard to most pre-treatment patient characteristics. However, the ones that completed might have experienced somewhat more relational difficulties as measured with personality disorder criteria. Interestingly, amongst the 21 adolescents who dropped out, level of confidence in and expectancy for the treatment’s helpfulness (as rated by adolescents before starting treatment) was equally high among both groups. Others have found expectations of treatment to be lower in those who drop out, albeit among adult populations (Meyer et al., 2002; Martino et al., 2012; Taylor et al., 2012). When the therapy process was in its beginning at session three, there were differences in therapist-rated motivation and willingness to engage in therapy, which was found to be significantly lower in those who dropped out, confirming previous research findings among adult populations (e.g., Martino et al., 2012; Taylor et al., 2012).

The inconclusiveness regarding the specific pre-treatment patient characteristics of those who drop out of therapy may be related to the difficulties and inconsistencies of how dropout is defined, as O’Keeffe et al. (2018) have argued. However, it may also be the result of considering and studying these as isolated and independent aspects, ignoring the importance of the complex mutual influence that a therapeutic dyad exerts on each other and in turn on the therapeutic process. As such, the second aim of this study was to explore the therapeutic process of those who dropped out in terms of how it can be described in general terms, but moreover in terms of important underlying interaction structures that may shed light on some aspects of what goes on in the therapeutic encounter in the lead up to a young person dropping out of therapy. Whilst the early sessions were found to be characterised as showing an overall good and collaborative working relationship between therapist and adolescent, exploring the APQ for underlying, explanatory factors revealed three distinct types of interaction structures, supporting evidence of the multidimensional nature of those who go on to drop out (e.g., Fiester, 1977; O’Keeffe et al., 2019). The first interaction structure was characterised by a mutually trusting and collaborative dyad, the second by an emotional detachment between both and a resistance of the adolescent to engage, and the third by a marked mismatch and incongruence in perception and communication between therapist and adolescent.

The identification of these three different types of interaction structures appear to support previous studies in that some patients, whether adults or adolescents, may leave therapy prematurely even if there is a good therapeutic process, whereas others leave because of problems in the therapeutic relationship (Todd et al., 2003; Roe et al., 2006; Westmacott et al., 2010; Jung et al., 2013). One possible explanation for this may relate to how the therapist themselves manages their emotional responses to the patient. Ligiéro and Gelso (2002) found that negative countertransference, that is, the emotional reactions of the therapist due to the patients’ projections, and poor therapeutic alliance were among the most frequent reasons for patients’ drop out in adults.

### Mutual Trust, Collaboration, and the Exploration of Emotions

The first interaction structure identified in this study was characterised by a mutually trusting and collaborative relationship between therapist and adolescent, where the adolescents felt held and confident enough to explore their thoughts and their painful past experiences of loss and current internal emotional states. In sessions from the IMPACT study, Calderón et al. (2019) found a similar interaction pattern in mainly STPP sessions which they named “Strong working relationship between an emotionally involved young person and a therapist who invites the young person to reflect on experiences and develop self-understanding.” For sessions characterised by this interaction structure, the therapy was relatively unstructured, and the therapist did not provide direct assurance or guidance. The young person felt understood and seemed to gain new understanding. In these sessions, the adolescent appeared to feel comfortable beginning and ending the sessions and did not express negative feelings toward the therapist. The similar mean scores on the WAI between therapist and adolescents of the third session of therapies which showed this interaction structure highlight a congruent perception of their therapeutic alliance that can be described as positive. In a study on countertransference, Ulberg et al. (2013) found that the alliance as rated by the therapist showed a positive relation to a feeling of confidence at the therapist’s part, which again may contribute to a positive experience for the adolescent.

Although results must be considered with caution due to limited data available, for those four patients with existing outcome data in this interaction structure, there appeared to be an improvement in both psychodynamic and global functioning, and a move from the moderate to the mild depression range on both the BDI and the MADRS. As such, one could tentatively wonder if these individuals ended therapy early because they felt sufficiently helped and better off after about six sessions of STPP including transference work (the one in this group that did not receive TW, did not come for follow-up interviews). At baseline, these adolescents showed low levels of personality pathology, high levels of symptoms, and in the therapy itself they seemed to respond very well to the therapist’s interventions; increasing their level of psychodynamic functioning after only a few sessions. The findings specifically mirror the dropout type ‘got-what-they wanted’ identified by O’Keeffe et al. (2019) among the depressed adolescents in the IMPACT study, who left therapy early because they felt satisfied and sufficiently helped by therapy, even if they hadn’t completed the whole therapy, and whose outcomes were comparable to those who completed therapy.

### Resistance and Emotional Detachment

O’Keeffe et al. (2019) distinguished between ‘got-what-they-needed’ and ‘dissatisfied’ dropouts, with only the latter group showing poorer outcomes than those who completed therapy. In line with O’Keeffe et al.’s findings, exploring the psychotherapy process in this study revealed two distinct types of patients whose interaction structures during sessions indicated that they may have left because they were dissatisfied.

The second interaction structure identified, which accounted for as much to the total variance as the first one, was characterised not only by an emotional detachment between therapist and young person, but furthermore by an absence of a discussion of the young person’s affect, including their emotional vulnerability. Most importantly, the adolescents in these sessions did not appear to be committed to the work of therapy and resisted all attempts of the therapist to engage. Consequently, the adolescents did not appear to feel helped, and they also expressed negative feelings toward the therapist. Calderón et al. (2019) found an interaction structure describing a similar dynamic, which they named “Difficult working relationship between a non-engaged young person and a therapist working hard to make sense of the young person’s experiences, but without making much progress.” Whereas the CBT and STPP therapists in Calderón et al.’s study worked toward making sense of the adolescents’ experience, asked for more information, and structured the sessions, the therapists characterised by the second interaction structure in the present study rather focussed on their young patients’ symptoms, encouraged them to discuss assumptions behind their experiences, and refrained from taking position in relation to their thoughts and behaviour. It would be of interest to see if these differences in therapist behaviour may be promoting dropout. The scores on the WAI, both patient and therapist-rated, further reflect a difficult working relationship, as overall mean scores for both were lower compared to the dyads characterised by the first interaction structure.

Of further interest, is that the current interaction structure was the only one that included a 50% split in gender and in which all adolescents had a comorbid anxiety disorder. They also appeared to differ from the others in that they had lower pre-treatment depression scores but on average also lower scores on the friendship and insight dimension on the PFS, characterised by a tendency to devaluate others and fearing being trapped or rejected, as well as a tendency for little reflection on personal motives and a denial to see symptoms as signs of disturbance. Within adult populations, low intrapsychic functioning was found to be a predictor of dropout (Rubin et al., 2018), whilst high intrapsychic functioning related positively to treatment engagement (Barrett et al., 2008). Moreover, the former has been empirically linked to poorer therapeutic alliance (Hersoug et al., 2009). Intriguingly, the identified interaction structure illustrates how such a dynamic can play out between therapist and patient. Although in two thirds of these therapies the therapist was asked to refrain from working in the transference, the therapists appeared to not display or show any emotional reaction toward the young person when trying to accommodate. This in turn might have made the young person feel more wary and suspicious of the therapist who may have appeared rather cold and distant, thereby promoting feelings of rejection. This seems to align with findings from von Below (2020) reported in the paper “We just did not get on,” based on young adults’ experiences of unsuccessful psychodynamic psychotherapy.

Furthermore, this interaction structure bears striking similarities to one of the alliance rupture types identified by Eubanks et al. (2019), namely withdrawal ruptures, in which the patient moves away from genuine engagement with the therapist and the therapeutic work. It is marked by avoidance, incongruent emotional display, minimal response, and refuting feeling states and events or relationships that seem significant to the therapeutic work. Addressing the relationship in the here-and-now, with the aim of repairing small ruptures proactively, is thought to prevent dropout (Safran and Muran, 2000). Ulberg et al. (2021) have found that adolescents are unlikely to talk about the relationship with the therapist on their own accord but may, if aided, share their thoughts and feelings of the therapeutic relationship and setting. Perhaps the young persons in sessions characterised by this interaction structure, suffered a lack of rupture-resolutions as their therapists refrained from addressing the affects and emotions in the room. This may, in turn, have contributed to the premature ending of therapy. Young people in this interaction structure, however, did try and address their unhappiness with the therapist, and in this instance, it was the therapists who seemed unable to bring it up. Considering the observations that the adolescents in this interaction structure had somewhat higher levels of personality pathology and lower levels of psychological functioning, it may be that these adolescents’ dysfunctional relational dynamic were recreated within the therapy setting. Tanzilli et al. (2020) found that higher levels of psychological functioning among adolescent patients were negatively related to countertransference reactions such as disengaged/hopeless, angry/criticised, disorganised/frightened, and overinvolved/worried. In adult populations, research has shown that there are important interactions between transference work, patient pathology, countertransference (Nissen-Lie et al., 2020) and outcome (Dahl et al., 2017). We can merely speculate that negative countertransference reactions were set in motion in this interaction structure, hampering with the therapist’s ability to be open and responsive. To our knowledge, no study thus far has explored the role of transference-countertransference patterns in promoting therapy dropout among adolescents. In fact, Kächele and Schachter (2014) argue that the most neglected factor in the study of psychotherapy dropout is the countertransference. Psychodynamic theory stresses that whilst therapists’ emotional reactions (or the lack of them) to the patient may facilitate understanding and formulation of the core problems, it has a significant impact on the therapeutic process (Winnicott, 1949) and may prove to be an obstacle for a good alliance and productive work if not monitored (Holmqvist, 2000) and managed adequately through supervision (Hayes et al., 2012). Ligiéro and Gelso (2002) found that negative countertransference patterns and poor therapeutic alliance were among the most frequent reasons for patients’ drop out.

### Mismatch and Incongruence in Perception and Communication

Albeit much smaller and less prevalent, the third interaction structure identified described yet another type of an unhelpful dynamic between therapist and adolescent. It is characterised by a mismatch and incongruence in perception, which appears driven by the therapist. Whilst the young person in sessions characterised by this type of interaction structure seemed committed to the work and displayed a capacity to link mental states, the therapist did not facilitate the young person to speak and did not encourage reflection. They overall seemed to adopt a rather authoritative, advisory but also judgemental approach. Consequently, the young person appeared to feel threatened and punished, unfairly treated as well as rejected and abandoned. The young person seemed unable to voice and address their concerns and feelings with the therapist. Keeping in mind the possible unrecognised countertransference feelings here too, it is of interest that the therapist seems to have abandoned the psychodynamic work with these adolescents altogether and adopted a structural and behavioural approach, which appears, however, not what the young person needs. Overall, individuals with sessions belonging to this interaction type attended more sessions than those in the other groups (9 compared with 7 and 5) before dropping out and were given the highest therapist-rated motivation score. However, the discrepant ratings between patient-rated and therapist-rated therapeutic alliance underscores the observed incongruence in perception of what is needed and communication between both.

Studies with adult populations in short-term treatment have shown that a lack of agreement between therapist and patient in terms of the formulation of the core problem, goals and how to achieve these increased premature dropout (Gabbay et al., 2003; Westmacott et al., 2010). Moreover, as Philips et al. (2018) have pointed out, whilst the well-established therapeutic ingredients of empathy, warmth and positive regard usually contribute to patients staying in therapy, negative responses, which include hostility, the adoption of an authoritarian or imposing stance and not allowing space for negative affect to be expressed and explored, have been associated with higher dropout rates (Mahon et al., 2001; Ogrodniczuk et al., 2005). Interestingly, in their study Philips et al. (2018) found that the therapists in the dropout group gave more explicit advice and guidance and behaved in a teacher-like manner, which is not dissimilar to how the therapist in the third interaction structure appeared to react.

### Limitations and Future Research

The present findings need to be considered within the context of several limitations. The first pertain to the methodological choices made. As already mentioned, there currently exists no consensus on how dropout is best defined and it remains one of the biggest challenges in studying it (Jung et al., 2013). The present study decided to follow the protocol which *a priori* decided that endings up to the 12th session were dropouts (Ulberg et al., 2012). New definitions, like the need-based definition of therapy dropout (Dossett and Reid, 2020), should be thoroughly explored in research. The findings raise questions about treatment dosage and a one-size-fits-all approach within mental health care services. A second major limitation is the small sample size which precluded the carrying out of test of differences between the dropout groups. As Rost (2021) has pointed out, one of the most difficult practical aspects to navigate when Q and R methodology and statistics are combined is around the sample size. For a Q-study a small sample size (i.e., number of Q-sorts) is not considered a problem (Smith, 2001), however, for subsequent group comparisons following R-statistics, it is often too small and underpowered. Irrespective of our definition of dropout, lack of available data reduced the sample size from 21 to 16 adolescents for whom we had reliable APQ ratings. The Q-analysis identified three sessions as confounders, which reduced the overall sample size even further. A further problem that is not unique to this study, was the missing data of dropouts. Therefore, although we did report outcome data to supplement the sense-making of the three types of interaction structures, these must be viewed tentatively, especially the comparisons between the groups. Furthermore, at this stage, we do not know to what degree the interaction structures identified are unique to dropout cases or could characterise early sessions for all depressed adolescents in the FEST-IT study. The comparison with the completers, which is currently under way and will be the subject of a separate paper, aims at shedding some light onto this.

Having said that, the present study aimed at being explorative and thereby hypothesis-generating in the hope that further research might replicate our findings as well as test these hypotheses more systematically in a larger sample of depressed adolescents. Future research might also open the investigation to include adolescents from different cultural and ethnic backgrounds. Almost all participants were from a white Norwegian origin and as such findings cannot be generalised to other cultural and ethnic groups. This is pertinent as patients being from an ethnic minority background have a higher risk of treatment dropout than ethnic majority patients and that dropout rates are ethnically specific (de Haan et al., 2018). Furthermore, in light of our observations regarding personality and psychological functioning, future studies ought to empirically examine these matters and their potential role in promoting therapy dropout among adolescents with larger samples.

A further limitation was the lack of any therapist variables to complement the sense-making and understanding of the interaction structures. Previous research has in particular highlighted that dropout rates were higher in treatments conducted by less experienced therapists (Swift and Greenberg, 2012). It would have thus been interesting to see if therapists differed in terms of their experience between those in the first compared to the second and third interaction group. A further important factor that led to drop out in the study carried out by O’Keeffe et al. (2019) were significant external challenges that provided a lack of stability for some young people to be able to engage in their therapy. We did not have any data on such possibilities, but it would have been interesting to see if some adolescents were dealing with such problems, especially those in the third interaction type. If so, it may have explained as to why the therapists adopted a more structured and solution-focussed approach in the session, if they felt there was not enough external stability for a more exploratory, psychodynamic approach.

A further major limitation of the current study is the fact that the therapy process was investigated only cross-sectionally and not over time. Also, maybe the use of video-recordings would have shed light on significant non-verbal communicative cues that are missed when using only audio-recordings. The decision to rate one session only was primarily driven by pragmatic reasons, however, for a fuller and deeper study of the therapeutic process and emerging dynamic between therapist and adolescent, future research should aim to rate the APQ for all available sessions. This would have not only allowed for an exploration of underlying interaction structure that account for the change and possible development of the dynamic over time, but moreover would have allowed to investigate empirically whether the formation of early interaction structures relate to later drop out. As Serralta (2016) has shown in her study, the modes of interaction structures identified early on in treatment were repeated over the course of the psychodynamic therapy. Her findings are important in highlighting the importance of setting up the right dynamic and interaction structure early on in treatment. Finally, it would have been an important addition to compare the current findings to both the overall description of treatment characteristics as well as possible interaction structures of those who completed treatment. However, this analysis is currently underway and will be the subject of a separate paper.

## Conclusion

The present findings have added to the growing research evidence that the reasons as to why adolescents with depression drop out of treatment prematurely are multidimensional. The findings, especially if replicated in a larger sample, may have important clinical implications. Understanding what happens early on in treatment between therapist and the young person, particularly in terms of what interaction structure is being formed and possibly developed throughout treatment, is crucial to mitigate premature dropout of those who have not felt helped and left dissatisfied and disappointed. This study has highlighted the importance of paying attention to the underlying dynamic relationship between therapist and young person and draws attention to the fact that its different manifestations can lead to different reasons for early dropout. Not all adolescents may leave early because they are dissatisfied; they may also leave because they feel sufficiently helped. The interaction structures identified in the present study clearly showed one configuration of mutual trust, collaboration, and enjoyment in the psychodynamic work. Others may leave because the dynamic and interaction structure between therapist and patient was not optimal from the beginning, hence we need to pay attention to these processes right from the first session onward to avoid unsatisfactory dropout. Despite its limitations, the present study has contributed with some important insight into the phenomenon of adolescent dropout from STPP.

## Data Availability Statement

The raw data supporting the conclusions of this article will be made available by the authors, without undue reservation.

## Ethics Statement

The study involving human participants was reviewed and approved by The Regional Committees for Medical and Health Research Ethics. Written informed consent from the participants’ legal guardian/next of kin was not required to participate in this study in accordance with the national legislation and the institutional requirements.

## Author Contributions

HF, H-SD, RU, JA, HJ, LS, and LT collected data for the present study. HF did the data analysis and wrote the initial draft of this manuscript in collaboration with and under the supervision of FR and H-SD. RU was Principal Investigator of the FEST-IT study and were responsible for the funding. RU and H-SD administered the project. NM and AT helped with conceptualizing the FEST-IT study. All authors have contributed, read, and approved the submitted version of the manuscript.

## Conflict of Interest

The authors declare that the research was conducted in the absence of any commercial or financial relationships that could be construed as a potential conflict of interest.

## Publisher’s Note

All claims expressed in this article are solely those of the authors and do not necessarily represent those of their affiliated organizations, or those of the publisher, the editors and the reviewers. Any product that may be evaluated in this article, or claim that may be made by its manufacturer, is not guaranteed or endorsed by the publisher.
